# Visceral urate deposition in a little bittern (*Ixobrychus minutus*)

**Published:** 2015-06-15

**Authors:** Morad Rahimi, Zahra Minoosh, Siavosh Haghighi

**Affiliations:** 1 *Department of Clinical Sciences, Faculty of Veterinary Medicine, Razi University, Kermanshah, Iran; *; 2 *Department of Pathology, Faculty of Veterinary Medicine, Razi University, Kermanshah, Iran.*

**Keywords:** *Bittern*, *Ixobrychus minutus*, *Urate deposition*, *Visceral gout*

## Abstract

Visceral urate deposition (visceral gout) is a common finding during post-mortem examination of poultry. Rare cases of visceral gout may occur in wild birds. A rare case of visceral urate deposition in a little bittern (*Ixobrychus minutus*) is reported here. In May 2013, carcass of a little bittern was submitted for necropsy to the Clinic of Poultry Diseases (Faculty of Veterinary Medicine, Razi University) by local authorities of Iran Department of Environment. At necropsy, white chalky deposits were observed on the heart and thoracic air sacs of the bird. To confirm the presence of urates, chalky deposits were collected from pericardium and tested by muerxide test. Heart and kidneys were sampled, preserved in 10% neutral-buffered formalin solution and submitted to laboratory for histopathology. Murexide test was positive for presence of uric acid in chalky deposits collected from pericardium. Light microscopy of affected organs confirmed the condition as visceral urate deposition. To the best of our knowledge, this is the first report on the occurrence of visceral urate deposition in a little bittern.

## Introduction

Gout is correctly used as a term in human medicine to describe an enzyme defect that causes an abnormal nitrogen metabolism resulting in excessive uric acid production. In avian medicine, “gout” is a historical misnomer whereas urate deposition is a more correct term.^1^ Visceral urate deposition (visceral gout) is defined as the accumulation of urates in kidneys, on serous surfaces of the heart, liver, mesenteries, and air sacs. In severe cases, surfaces of muscles and synovial sheaths of joints may be involved, and precipitation may occur within the liver, spleen, and other organs. The deposits on serous surfaces appear grossly as a white chalky coating, while those within visceral organs may only be recognized microscopically.^2^ The patho-genesis of visceral urate deposition is not completely clear, but it is generally associated with conditions that reduce uric acid excretion or increase uric acid production.^3^^-^^5^

Pathologic examination of gouty lesions confirms the diagnosis of visceral urate deposition by demonstrating urate tophi.^6^^,^^7^ Visceral urate deposition has been reported in various caged and aviary birds from different parts of the World. It is among the most commonly diagnosed causes of mortality in poultry.^8^^,^^9^ Rare cases of visceral urate deposition may occur in wild birds. A rare case of visceral urate deposition in a little bittern (*Ixobrychus minutus*) is reported here.

## Case Description

**Fig. 1:**
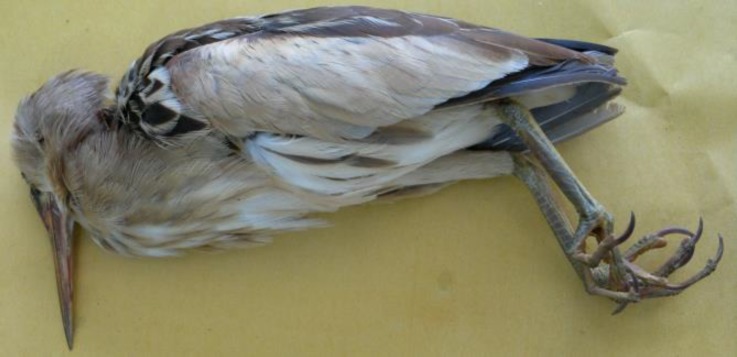
Carcass of the little bittern referred by local authorities of Iran Department of Environment for necropsy.

In May 2013, carcass of an adult female little bittern (Fig. 1) was submitted for necropsy to the Clinic of Poultry Diseases (Faculty of Veterinary Medicine, Razi University) by local authorities of Iran Department of Environment. The bird was found moribund by an environment watchman of Hashilan wetland. Hashilan, one of the habitats of little bittern, is a 260-hectares freshwater wetland, located at 35 km northwest of Kermanshah, west of Iran.^10^ No treatment had been done, and the bird had died just before submission to the clinic.

**Fig. 2:**
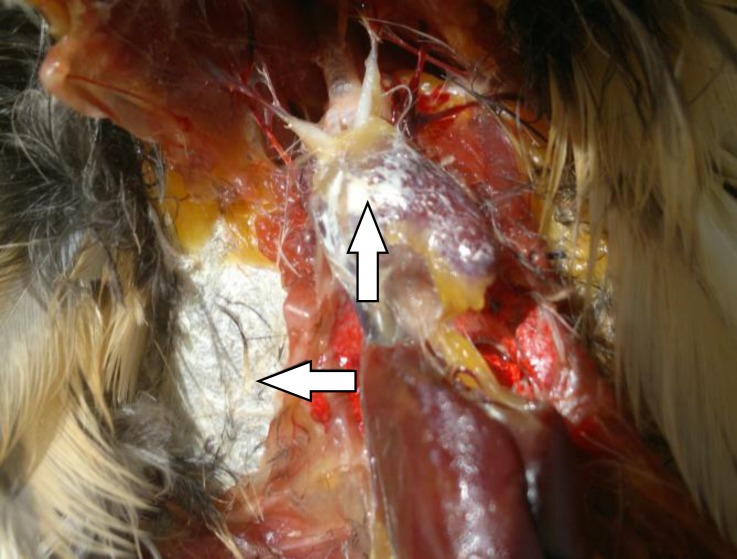
Urate deposits (arrows) on the heart, and thoracic air sacs of a little bittern with visceral urate deposition

Routine necropsy was performed on the dead bird. White chalky deposits were observed on serous surfaces of the heart and thoracic air sacs (Fig. 2). No obvious gross lesions could be detected in kidneys and other visceral organs. To confirm the presence of urates, chalky deposits collected from pericardium were tested by muerxide test as described.^6^ Briefly, a drop of nitric acid was mixed with scant amount of deposits on a slide which was slowly flame dried. After drying, the slide was cooled at room temperature and a drop of ammonia was added to the mixture.

Heart and kidneys were sampled, preserved in 10% neutral-buffered formalin solution and submitted to laboratory for histopathology. The tissues were then processed, embedded with paraffin, sectioned, and stained with hematoxylin and eosin (H & E) for light microscopy.

**Fig. 3:**
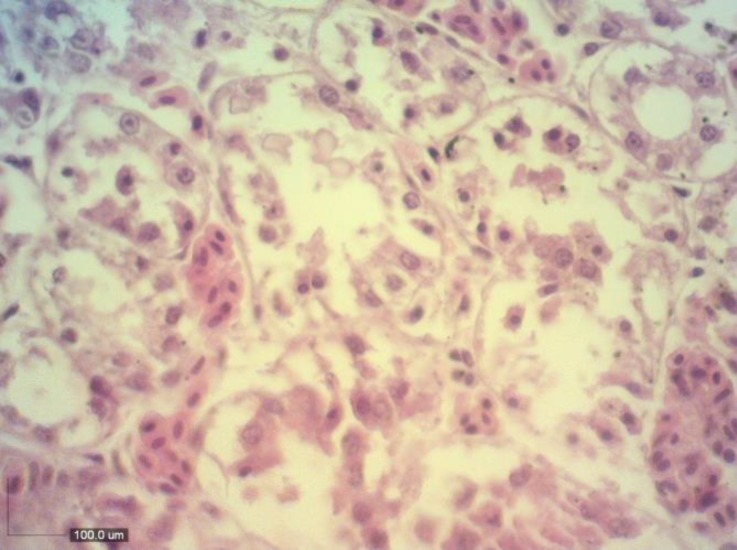
Micrograph of kidney indicating severe tubular epithelial cell degeneration and necrosis in a little bitternwith visceral urate deposition (H & E, 400×).

**Fig. 4:**
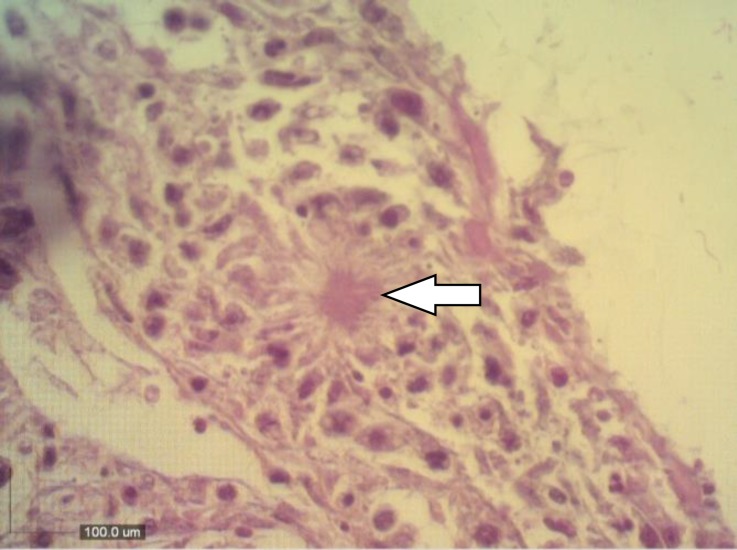
Pericarditis with urate tophi (arrow) in a little bitternwith visceral urate deposition (H & E, 400×).

Appearance of light purple color in murexide test indicated the presence of urates in chalky deposits. This test can be used to confirm the presence of urates in deposits on the surfaces of visceral organs.^6^ At light microscopy, kidneys were physically damaged by urate deposition, and scant amount of inflammatory cells infiltration was observed. Severe tubular epithelial cell degeneration and necrosis were noted (Fig. 3). Histopathological examination of the heart revealed extensive pericarditis with urate tophi in both pericardium (Fig. 4) and myocardium along with muscle fibre necrosis.

## Discussion

Visceral urate deposition is a sign of severe renal dysfunction that causes hyperuricemia.^7^ In poultry, apart from renal failure, dietary protein above the bird requirements may also cause hyperuricemia.^11^ Dehydration due to water deprivation is a common cause of visceral urate deposition in domestic poultry. Outbreaks of visceral urate deposition in poultry have also been attributed to infectious causes, such as nephrotropic strains of infectious bronchitis virus^12^ and renal crypto-sporidiosis;^13^ and non-infectious factors, such as vitamin A deficiency, secondary to urolithiasis,^5^ treatment with sodium bicarbonate,^14^ and mycotoxins, such as oosporein.^15^

Masses of fat deposits in peritoneal cavity of the little bittern and relatively good musculature showed that the condition had led to its death during an acute course. In acute renal failure visceral urate deposition might occur alone.^11^ Inflammatory reactions are not often detected as birds die rapidly. Hyperkalaemia can develop, and this, rather than the uric acid, might lead to cardiac arrest and the sudden death seen with visceral urate deposition.^7^ Factors, such as dehydration and vitamin A deficiency are common causes of visceral urate deposition in domestic poultry,^5^ but they could not cause visceral urate deposition in domestic poultry,^5^ but they could not cause visceral urate deposition and acute death in this case, since little bitterns had free access to water sources and variety of feeds.

Although it is still theorized that long term of high-protein feeding may induce hyperuricemia in granivorous or nectivorous birds,^4^^,^^16^ Guo *et al.* induced visceral urate deposition in growing layers fed high calcium and high protein diets.^17^ As little bitterns are essentially insectivorous and take aquatic adult and larval insects, molluscs, crustaceans, fish, frogs, tadpoles, small reptiles and birds,^18^ they are adopted to high protein and high calcium diets. As a result, high protein and high calcium diets are unlikely to cause visceral urate deposition in little bitterns.   

In conclusion, visceral urate deposition and acute death in this little bittern might be due to acute intoxication. Because of recent droughts in Iran, especially in western parts of the country, little bitterns are threatened by habitat degradation and loss through direct destruction, pollution and hydrological changes in rivers, lakes and wetlands.
